# Visual Secure Image Encryption Scheme Based on Compressed Sensing and Regional Energy

**DOI:** 10.3390/e23050570

**Published:** 2021-05-06

**Authors:** Mengna Shi, Shiyu Guo, Xiaomeng Song, Yanqi Zhou, Erfu Wang

**Affiliations:** Electrical Engineering College, Heilongjiang University, Harbin 150080, China; shimengna008@163.com (M.S.); guoshiyubaby@163.com (S.G.); 18846433840@163.com (X.S.); zhouyanqi1998@163.com (Y.Z.)

**Keywords:** image encryption, compressed sensing, regional energy, multi-character chaotic system

## Abstract

The network security transmission of digital images needs to solve the dual security problems of content and appearance. In this paper, a visually secure image compression and encryption scheme is proposed by combining compressed sensing (CS) and regional energy. The plain image is compressed and encrypted into a secret image by CS and zigzag confusion. Then, according to the regional energy, the secret image is embedded into a carrier image to obtain the final visual secure cipher image. A method of hour hand printing (HHP) scrambling is proposed to increase the pixel irrelevance. Regional energy embedding reduce the damage to the visual quality of carrier image, and the different embedding positions between images greatly enhances the security of the encryption algorithm. Furthermore, the hyperchaotic multi-character system (MCS) is utilized to construct measurement matrix and control pixels. Simulation results and security analyses demonstrate the effectiveness, security and robustness of the propose algorithm.

## 1. Introduction

With the rapid developments of Internet and computer technology, digital network communication technology is widely used in various fields of society. While the open and sharing nature of the Internet bring convenience, it also poses some potential security problems. For example, data transmitted over the network is intercepted and leaked by illegal users, maliciously tampered, and false information is sent with a legitimate identity. These issues disrupt the network order and threaten people’s private information. As a visual representation of information carriers, digital images are frequently used in people’s daily network communication with their excellent visual characteristics. Therefore, image information security has become a social research hotspot in recent years.

Image encryption technology protects the data security of transmitted images by encryption algorithms that apply keys to plain images to generate another form of image irrelevant to the plain image. Traditional encryption algorithms generally convert image data into one dimensional data stream before encrypting it, ignoring characteristics such as the two-dimensional nature of the image and the large volume of data, making it difficult to meet the real-time requirements of transmission. To ensure secure and efficient transmission of images, plenty of schemes have been submitted that apply techniques such as chaotic system [1,2,3,4,5], DNA [6,7,8], optics [9,10,11], cellular automation [12,13], elliptic curve [14,15], and fuzzy [16] to image encryption. Among them, chaotic system is widely used in image encryption with excellent characteristics such as pseudo-randomness and initial value sensitivity. Liu et al. [1] designed a new 2D-SIMM hyperchaotic mapping that use chaotic sequence to shuffle the image rows and columns to scramble the correlation between adjacent pixels. Çavuşoğlu et al. [2] proposed an image encryption algorithm based on S-box chaos. A random number generator was designed by using the chaotic system, and the output sequence was used for the original image Bit XOR and S-box, respectively, and then the S-box was used for sub-byte processing of the XOR processed image to obtain the final secure encrypted image. Hua et al. [3] proposed a new compound chaotic system 2D-LSCM and applied it to image encryption. The algorithm uses the classical Scrambling–Diffusion framework for four rounds to ensure that the encryption algorithm is secure and efficient, but is not resistant to strong attacks. Wang et al. [4] proposed a new method of image encryption based on matrix half-tensor product theory, which used chaotic sequence scrambling and half-tensor product diffusion to give more forms to the encryption scheme. Huang et al. [5] proposed a symmetric color image encryption system. Simultaneous scrambling and diffusion make the encryption level higher, and the first encrypted pixel position is controlled by the original image and the key stream generated by the chaotic system. The algorithm is sensitive to the input image and provides better security against chosen-plaintext attack (CPA) and known-plaintext attack (KPA).

In the era of big data, there is a high demand for speed in image transmission and storage. Without affecting the visual effect of the image information, appropriate compression of the transmitted images can save transmission bandwidth and improve transmission efficiency. Compressed sensing (CS) technology can obtain discrete samples of sparse signals with a random observation matrix at a much lower sampling rate than Nyquist, and recover the signals approximately with a non-linear reconstruction algorithm. The advent of CS makes it possible to compress and encrypt images simultaneously. In [17], it has been proved that the projection measurement of CS was computationally confidential. Chen et al. [18] proposed an image encryption algorithm based on chaos and CS. The measurement matrix was generated by chaotic system, and the robustness of the encryption algorithm was enhanced by cascading CS and scrambling diffusion framework. Zhou et al. [19] combined 2D CS and nonlinear fractional Merlin transform to propose a new image encryption system, which has a larger key space compared than others. Chai et al. [20] proposed an image encryption algorithm based on memory chaotic system, cellular automata and compressed sensing. By adding zigzag obfuscation and calculating the relevant parameters and initial values with original images, the data storage capacity is reduced, and it has higher security and robustness. Hua et al. [21] proposed a chaotic magic transform based on 2D-SLMM to scramble image pixel positions, which reduced the time complexity and the compound chaotic system improved algorithmic security. Encryption algorithms similar to the above all result in meaningless images that are class noise or class texture. This algorithm effectively protects the data content of the plain image, but it is prone to attract the attention of third parties during transmission or storage, leading to focused attacks. Therefore, to enhance the security of the encryption algorithm and to guarantee the security of the encrypted image appearance, it is necessary to hide its presence in the channel.

Bao et al. [22] proposed the concept of encrypting the original image into a visually meaningful image and embedding the pre-encrypted image into the frequency domain sub-band of the reference image to generate an encrypted image with visual appearance. Based on this concept, Yang et al. [23] extended the new method of meaningful image encryption by using discrete wavelet transform and discrete computation to eliminate the texture features of the encrypted image. Gong et al. [24] proposed an effective image encryption scheme based on chaotic system and CS. Arnold transform was adopted to reduce the compressed block effect, and chaotic system key was associated with plain image. the algorithm provides excellent compressibility and security. Zhu et al. [25] proposed a robust and meaningful image encryption algorithm. The secret image was first encrypted as a secret image used CS, and singular value decomposition was used to embed the secret image into the carrier image to generate the final meaningful cryptographic image. The scheme allows parallel processing of medium and large images, improving the robustness and efficiency of the algorithm. Chai et al. [26] used zigzag obfuscation and CS to compress and encrypt the original image into a meaningless image, and adopted the LSB method to embed the carrier image to produce the final visually meaningful cipher image. Chai et al. [27] was split the secret image into an integer quotient matrix and a remainder matrix, and the two intermediate frequency matrices of the carrier image was replaced with the median of the low-frequency matrix of the carrier image as the threshold, and ultimately obtained a visually secure cryptographic image. The method has no additional transmission, but the direct replacement of the mid-frequency elements reduces the concealment of the secret image. The above-mentioned literatures conceal the existence of secret images by directly weighting or replacing the carrier images, and finally generate visually meaningful cipher images. However, the influence of the embedding position of the secret image on the destruction size of the carrier image is not considered. Therefore, it is necessary to choose a suitable embedding method to reduce the damage to carrier image.

In this paper, we present a method of hour hand printing (HHP) scrambling, and a new image visual security encryption algorithm based on CS and regional energy is introduced. The encryption process of the scheme is divided into two stages. In the first stage, the plain image is compressed and encrypted using compressed sensing technology and zigzag obfuscation, and then the noise-like secret image is generated by diffusion and HHP scrambling operation. In the second stage, the secret image is embedded in the carrier image depending on the regional energy, ultimately generating a visually secure cipher image. The optimal measurement matrix of compressed sensing is constructed by multi-character system (MCS), and the initial values of the chaotic system is related to plain image. In addition, chaotic sequences are used for bit-level and pixel-level scrambling operations. Simulation results and performance analysis verify the effectiveness and security of the proposed encryption algorithm.

The contributions of this paper are as follows.

(1) The HHP scrambling method is proposed to increase the randomness between elements and enhance the security of the encryption scheme. (2) The initial value of chaotic system is related to plain image, strengthening the immunity of the encryption algorithm to CPA and KPA. (3) By calculating the regional energy of the image, the optimal embedding position is found to reduce the damage of the secret image to the carrier image. (4) After the embedding position is determined, the embedding can be carried out in parallel to save time and efficiency.

The rest of this paper is organized as follows. Section 2 introduces the fundamental theories involved in encryption algorithm, including compressed sensing technology, multi-character hyperchaotic systems, and zigzag obfuscation. Section 3 introduces the hour hand printing method and embedding strategy based on region energy. Section 4 describes a detailed plan of the proposed encryption algorithm. Section 5 shows the simulation results. Section 6 analyzes the performance of encryption algorithm. Section 7 summarizes the whole work drawing conclusions.

## 2. Fundamental Knowledge and Related Technologies

In this section, the supporting theories and technologies relating to cryptographic algorithms are presented, including compressed sensing, multi-character chaotic system, and zigzag confusion.

### 2.1. Compressed Sensing

Compressed sensing (CS) as a novel signal sampling technology breaks through Nyquist sampling theorem. Its theoretical framework is mainly divided into three parts including the sparse signal representation, construction of measurement matrix and signal reconstruction [28,29]. The mathematical model of CS is formulated as if the one-dimensional discrete signal *x* of length *N* is sparse in the transformation domain Ψ, then *x* is called a compressible signal. The matrix form is as follows:

(1) $$x=\sum_{i=1}^{N}\Psi_{i}s_{i}=\Psi s,$$

where *s* is the N×1-dimensional coefficient vector representation of the signal *x* in the Ψ domain, and Ψ is the N×N-dimensional sparse basis of the signal *x*. When the number of non-zero coefficients in *s* is *k* (k≪N), then the signal *x* is called to be *k*-sparse in the Ψ domain.

After selecting the sparse basis matrix, using the matrix Φ that is uncorrelated with the sparse basis, to linearly project the signal *x* to obtain the linear measure *y*.

(2) y=Φx,

where Φ is a measurement matrix of size M×N (M≪N) and *y* is the measurement value matrix of with the size of M×N. By substituting Equation (1) into Equation (2), one gets.

(3) y=Φx=ΦΨs=Θs,

where Θ=ΦΨ is the sensing matrix of size M×N.

The reconstruction of the signal is the final step to complete the compressed sensing, that is, to recover the original signal *x* from *y*. In order to recover *x* accurately as possible, Θ is required to satisfy the restricted isometry property (RIP) [30]. Based on this condition, commonly used measurement matrices include Gaussian random matrix, part-Fourier matrix and part-Hadamard matrix, etc. [31]. Compressed sensing reconstructed signal can be achieved by solving the following objective functions.

(4) $$\hat{s}=\arg\min{\left\|s\right\|}_{lp}s.t.y=\Phi \Psi s,$$

where ${\left\|t\right\|}_{lp}$ denotes the $l_{p}$-norm of vector *t*. When *s* is obtained by solving (4), *x* is further reconstructed by x=Ψs. There are two classes of reconstruction algorithms in common use today. One is based on greedy iterative algorithms that seek to minimum the $l_{0}$ norm, including matching pursuit, orthogonal matching pursuit (OMP), etc. Another is convex optimization algorithms that translate to finding the $l_{1}$ norm, including base pursuit, gradient projection method, etc. [32]. In this paper, the OMP algorithm is applied to reconstruct the signal.

### 2.2. Multi-Character Chaotic System

The high-dimensional hyperchaotic system has a more complex structure than the low-dimensional chaotic system, and the randomness of the generated sequence is stronger, which is more suitable for image encryption [33]. The 4D multi-character system (MCS) is defined by [34].

(5) $$\left\{\begin{array}{c} \dot{x}=-ay\\ \dot{y}=bwz+r\\ \dot{z}=y^{2}-cz^{2}\\ \dot{w}=x+y-wz-yz \end{array}\right.,$$

where *x*, *y*, *z* and *w* are the state variables of the system, with *a*, *b*, *c* and *r* being the fixed parameters of the system. When a=0.05, b=5, c=0.28, and r=0.1, the system is in hyperchaotic state with no equilibrium point and hidden attractor. Hidden attractors are unpredictable and unable to be found by conventional methods. The four Lyapunov exponents of the chaotic system are $\lambda_{1}=0.0764$, $\lambda_{2}=0.0287$, $\lambda_{3}=0$, and $\lambda_{4}=-1.6772$. The four random sequences generated from this system will be used in the subsequent construction of measurement matrix and image scrambling.

### 2.3. Zigzag

**Figure 1:**
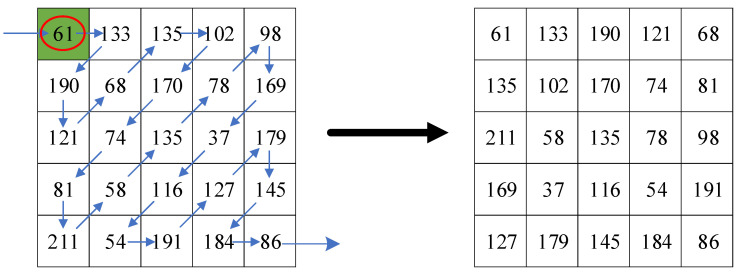
Path of zigzag confusion.

Zigzag confusion is the process of scanning the elements of a matrix in zigzag path to obtain a one-dimensional sequence, starting from the top left-hand element of the matrix, and converting it into a two-dimensional matrix in a certain way [35]. Using the Zigzag transform reduces the correlation between the sparse coefficients of a normal image. Since the non-zero coefficients in the sparse matrix are concentrated in the low frequency part of the matrix, to boost the quality of the image reconstruction, the low frequency components are allowed to appear earlier and the high frequency components later. Use zigzag confusion to rearrange the non-zero coefficients of a matrix into the top of a two-dimensional array. As shown in Figure 1, starting from the $\left(1,1\right)$ position of the left matrix, the matrix elements are scanned according to the sawtooth path, and the scanning direction is shown by the arrow in the left. Arrange the scanned elements into a new two-dimensional matrix, and the scan result is shown in the matrix on the right.

## 3. Hour Hand Printing Scrambling and Embedding Strategy Based on Regional Energy

In this section, a method of hour hand printing (HHP) for scrambling the position of image elements is proposed. In addition, the strategy for embedding the secret image into the carrier image is described based on regional energy. Embedding the secret image block into the carrier image block by combining optimal matching of energy values and coefficient weighting ultimately generates a visually cipher image for secure transmission in the channel.

### 3.1. Hhp Scrambling

In order to promote the uncorrelation between image pixels, we propose the HHP arrangement based on chaotic sequence index scrambling. The details are as follows.

To begin with, the two-dimensional matrix is stretched into a one-dimensional sequence, scrambled with the chaotic sequence index, and then arranged into the two-dimensional matrix as follows.

1. For odd circles, start from the top left position and arrange in a clockwise direction;
2. For even circles, start from the bottom right position and arrange in anti-clockwise direction;
3. Alternating odd and even circles until the two-dimensional matrix is filled, then the HHP is completed.

**Figure 2:**
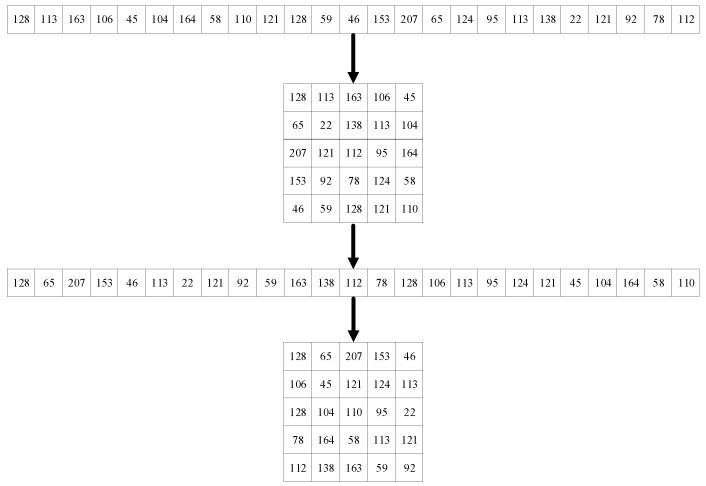
Two rounds of HHP.

Compared to traditional scrambling, HHP increases the randomness of the matrix elements and improves the security of the encryption algorithm. In this paper, the chaotic sequence index is used to disorder the one-dimensional vector first, and then the two rounds hour hand printing arrangement is performed. The results are shown in Figure 2. Once the first round of clockwise printing is complete, the first print matrix is re-stretched into a one-dimensional array in the columnar direction and the second round of clockwise printing is performed again.

### 3.2. Embedding Strategy Based on Regional Energy

Image energy is a measure of the grey value of an image, where the energy of a region of an image is the sum of the squared grey values of the image pixels in that region. The energy of a region is calculated as shown below:

(6) $$E=\sqrt{\sum_{x=1}^{m}\sum_{y=1}^{n}{\left(F(x,y)\right)}^{2}},$$

where F(x,y) represents the gray value of image at point (x,y) in which *m* and *n* are the *F*-region boundaries. In order to reduce the damage of the embedding process to the visual quality of the carrier image, a feasible solution is to embed the embedding area in the carrier area with similar energy.

The secret image is divided into different embedded regions, and the carrier image is divided into different carrier regions. According to Equation (6), calculate the energy of all embedded regions and embedded regions, and the energy of these two types of regions are sorted from smallest to largest, respectively, and the indexes of the two sets of sequences are regarded as a set of mappings. Depending on the corresponding position in this set of mappings, the embedding region is embedded in the carrier region in a weighted manner. To supplement an example showing the embedding process, set the energy of the embedding region to be (126,100,258) and the energy values of the carrier region to be (230,246,184) in order. In turn, their sorting and indexing are (100,126,258) corresponding to (2,1,3), and (184,230,246) corresponding to (3,1,2), respectively. The embedded region index (2,1,3) and the carrier region index (3,1,2) form a set of mappings. Thus, the embedding method is to weight embedding region 2 into carrier region 3, embedding region 1 into carrier region 1 and embedding region 3 into carrier region 2. Combining all the modified carrier regions results in a visually secure cipher image.

## 4. Encryption and Decryption Scheme for Visually Secure Images

In this section, we generate some vital values needed for the encryption scheme and then detail the specific encryption and decryption process.

### 4.1. Generation of Vital Values and Construction of Measurement Matrix

#### 4.1.1. Obtaining Initial Values of Multi-Character Chaotic Systems

This paper generates the initial values of chaotic system by calculating the mean and standard deviation of plain image, enhancing the ability of the encryption algorithm to withstand chosen-plaintext attack (CPA) and known-plaintext attack (KPA). Assume the size of the plain image *P* is N×N and the mean pixel value of *P* is expressed as follows.

(7) $$AV=\frac{\sum_{i=1}^{N}\sum_{j=1}^{N}P(i,j)}{N\times N},$$

The pixel standard deviation of *P* is expressed as follows.

(8) $$STD=\sqrt{\frac{\sum_{i=1}^{N}\sum_{j=1}^{N}{\left(P(i,j)-AV\right)}^{2}}{N\times N}},$$

where P(i,j) is the pixel value of the *i*-th row and *j*-th column of image *P*.

Perform floor(AV) and floor(STD) operations on the mean AV and standard deviation STD of image *P*, where floor(x) calculates the largest integer that is no more than *x*. Convert the rounded parameters into 8 bits of binary and divide them by 4 bits to obtain four 4-bit binary parameters, then convert the four parameters back to decimal as AV1, AV2, STD1 and STD2. For example, when floor(AV) is 97 and floor(STD) is 56, their 8-bit binary is “01100001” and “00111000”. Divided by 4 bits and then converted to decimal AV1=6, AV2=1, STD1=3, STD2=8 can be obtained.

In this paper, a 4D multi-character system (MCS) is used to construct the measurement matrix of compressed sensing and perform scrambling and disturbing operation. The initial value of 4D MCS is calculated as follows.

(9) $$\left\{\begin{array}{c} x_{0}=\frac{AV1}{15}+t_{1}\\ y_{0}=\frac{AV2}{15}+t_{2}\\ z_{0}=\frac{STD1}{15}+t_{3}\\ w_{0}=\frac{STD2}{15}+t_{4} \end{array}\right.,$$

where $t_{1}$, $t_{2}$, $t_{3}$ and $t_{4}$ are external parameters.

#### 4.1.2. Construction and Optimization of Measurement Matrix

Chaotic system construction measurement matrix can greatly reduce the number of keys for CS encryption. The MCS is iterated to $l_{0}+MNd$ times with the initial values $x_{0}$, $y_{0}$, $z_{0}$, $w_{0}$ and the control parameters *a*, *b*, *c*, *r*, where *d* is the sampling distance. To avoid transient effects, the former $l_{0}$ values are discarded. Using *d*-interval distance sampling, four random sequences $X={\left\{X_{i}\right\}}_{i=1}^{MN}$, $Y={\left\{Y_{i}\right\}}_{i=1}^{MN}$, $Z={\left\{Z_{i}\right\}}_{i=1}^{MN}$ and $W={\left\{W_{i}\right\}}_{i=1}^{MN}$ with a size of 1×MN are obtained. Obtained the new sequence $R={\left\{R_{i}\right\}}_{i=1}^{MN}$ by the following equation.

(10) $$R_{i}=\frac{X_{i}+Z_{i}+W_{i}}{3},i=1,2,\ldots ,M\times N,$$

where $X_{i}$, $Z_{i}$, $W_{i}$ are the *i*-th element of the *X*, *Z*, *W* sequence, respectively.

Sort the *Y* sequence and mark the generated index sequence as IdY, and IdY to sort and scramble the sequence *R* to obtain a sequence $R^{\prime }$ of size 1×MN. Arrange $R^{\prime }$ as follows to construct a chaotic measurement matrix Φ of size M×N.

(11) $$\Phi =\left(\begin{array}{c} R{}_{0}^{\prime }\\ R{}_{1}^{\prime }\\ \vdots \\ R{}_{M-1}^{\prime } \end{array}\right.\begin{array}{c} \cdots \\ \cdots \\ \vdots \\ \cdots \end{array}\left.\begin{array}{c} R{}_{M(N-1)}^{\prime }\\ R{}_{M(N-1)+1}^{\prime }\\ \vdots \\ R{}_{MN-1}^{\prime } \end{array}\right),$$

For improving the image reconstruction quality, the measurement matrix Φ is optimized by singular value decomposition (SVD), which can be expressed as Φ=U·S·V. where *U* and *V* are two orthogonal matrices and $S=\left[\begin{array}{c} S_{1}\\ 0 \end{array}\right.\left.\begin{array}{c} 0\\ 0 \end{array}\right]$ is the diagonal matrix. $S_{1}=diag\left(\sigma_{1},\sigma_{2},\cdots ,\sigma_{M}\right)$ contains the singular values of the matrix Φ and represents the significant features of matrix Φ, and $\sigma_{1},\sigma_{2},\cdots ,\sigma_{M}$ is arranged in descending order. Calculate the mean value $\sigma^{\prime }$ of diagonals of $S_{1}$, and obtain ${S_{1}}^{\prime }=diag\left(\underset{M}{\underset{︸}{\sigma^{\prime },\sigma^{\prime },\cdots \sigma^{\prime }}}\right)$ by $\sigma_{1}=\sigma_{2}=\cdots =\sigma_{M}=\sigma^{\prime }$, giving a new diagonal matrix $S^{\prime }=\left[\begin{array}{c} {S_{1}}^{\prime }\\ 0 \end{array}\right.\left.\begin{array}{c} 0\\ 0 \end{array}\right]$. Optimized measurement matrix $\Phi^{\prime }$ of size M×N is obtained by $\Phi^{\prime }=USV^{T}$.

### 4.2. Visual Security Image Encryption Algorithm

**Figure 3:**
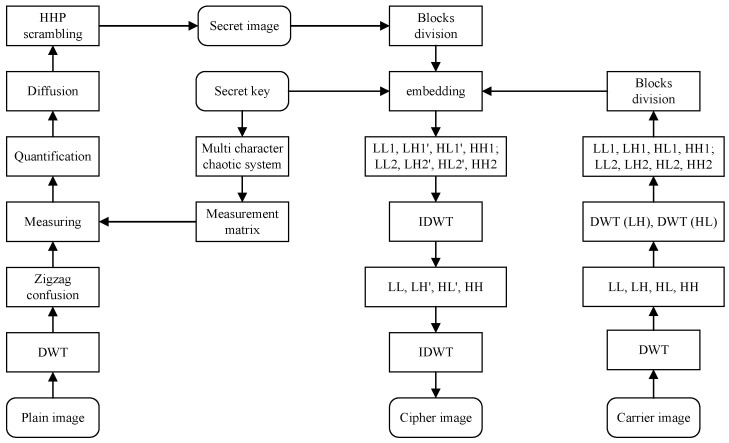
Encryption flow chart of the proposed algorithm.

In this section, a visual secure image encryption algorithm based on CS and region energy is proposed. The encryption algorithm consists of two phases and the encryption flow diagram is shown in Figure 3. In the first stage, the plain image *P* is compressed and encrypted into noise-like secret image $C_{se}$ utilizing compressed sensing, MCS, zigzag confusion, Diffusion and HHP scrambling methods. In the second stage, the region energy is used to control the embedding position and the secret image is embedded into carrier image, eventually acquiring a visually meaningful cipher image $C_{en}$.

#### 4.2.1. Compressing and Encrypting Plain Image into a Secret Image

Suppose a plain image *P* of size N×N is compressed and encrypted to create a secret image $C_{se}$ of size M×N. The specific steps are as follows:

**Step 1:** Use discrete wavelet transform (DWT) to perform sparse transformation on image *P* determining a matrix of sparse coefficients of the same size P1. As described in Section 2.3, Zigzag confusion is performed on the P1 matrix to obtain the P2 matrix. Set a threshold TS and modify the values in P2 that are less than TS to 0.

**Step 2:** Set a compression ratio as CR, According to the description in Section 4.1.1 and Section 4.1.2, calculate the initial values of the MCS, and then the measurement matrix Φ of size M×N is constructed, and Φ is optimized by SVD to obtain $\Phi^{\prime }$, where M=CR×N.

**Step 3:** Perform a linear projection measurement of the P2 matrix with $\Phi^{\prime }$ to obtain a measurement values matrix P3 of size M×N, that is $P3=\Phi^{\prime }P2$.

**Step 4:** Quantize the element of matrix P3 to the range [0,255] according to the following formula to obtain the matrix P4.

(12) $$P4\left(i,j\right)=floor\left(255\times \frac{P3\left(i,j\right)-P3_{\min}}{P3_{\max}-P3_{\min}}\right),$$

where P3(i,j) and P4(i,j) are the elements of row *i* and column *j* of the matrices P3 and P4, respectively. $P3_{\max}$ is the maximum value of P3, and $P3_{\min}$ is the minimum value of P3.

**Step 5:** Perform the diffusion operation on the matrix P4 by following these steps.

**Step 5.1:** Convert the *Y* sequence of size 1×MN in Section 3.2 to an integer by the following equation to obtain the sequence Y1 in the range [0,255].

(13) $$Y1\left(i\right)=\;\mathrm{mod}\;(floor\left(Y\left(i\right)\times 10^{14}\right),256),$$

**Step 5.2:** Stretch the P4 matrix to the size of 1×MN and perform pixel diffusion according to Equation (14) to obtain matrix P5 of size 1×MN.

(14) P5(i)=P4(i)⊕Y1(i)⊕P5(i−1),i=1,⋯,MN,

Advance setting P5(0)=floor(AV).

**Step 6:** Note that the index sequence after sorting the *R* sequence in Section 4.1.2 is IdR. According to Section 3.1, use IdR to scramble P5 to obtain a matrix P5 of size 1×MN. Run two rounds of HHP on matrix P6 to obtain a secret image $C_{se}$ of size M×N.

#### 4.2.2. Embedding the Secret Image into Carrier Image

Assume that the size of carrier image *D* is 2N×2N, but the size of carrier image is not limited to *D* is 2N×2N, which mainly depends on the compression rate CR. The smaller the CR, the smaller the carrier size can be selected. To ensure high visual quality and safety requirements, the size of the carrier image should not be smaller than the size of the plain image.

**Step 1:** To avoid overflow of the carrier image pixel values after embedding, adjust the carrier image pixel values to the [5,242] range according to the following formula.

(15) D1(i,j)=floor(5+0.949×D(i,j)),

where D(i,j) and D1(i,j) represent the grayscale values of original carrier image and modified carrier image, respectively.

**Step 2:** Perform DWT on D1 to obtain LL, LH, HL, HH matrices all of size N×N.

**Step 3:** Perform DWT on matrices LH and HL, respectively. generates four matrices LL1, LH1, HL1 and HH1 of size N/2×N/2, and HL generates four matrices LL2, c and HH2 of size N/2×N/2.

**Step 4:** According to Algorithm 1, the secret image is embedded into LH1, HL1, LH2 and HL2 matrices of carrier image, and modified matrices are noted as $LH1^{\prime }$, $HL1^{\prime }$, $LH2^{\prime }$ and $HL2^{\prime }$.

**Step 5:** Apply the inverse DWT on $\left(LL1,LH1^{\prime },HL1^{\prime },HH1\right)$ and $\left(LL2,LH2^{\prime },HL2^{\prime },HH2\right)$ to obtain $LH^{\prime }$ and $HL^{\prime }$ matrices, respectively. Apply the inverse DWT on $\left(LL,LH^{\prime },HL^{\prime },HH\right)$ to generate a visually security cipher image $C_{en}$.
**Algorithm 1** The embedding process.**Input:** The secret image $C_{se}$, four matrices LH1, HL1, LH2 and HL2 of the carrier image, embedding intensity factors *a*.**Output:** a set of mappings recording the embedding positions, four modified matrices $LH1^{\prime }$, $HL1^{\prime }$, $LH2^{\prime }$ and $HL2^{\prime }$.(1): The secret image $C_{se}$ is divided into non-overlapping blocks, each block is represented by $C_{se\_b}\left(i\right),i=1,\cdots ,T$, and *T* is the sum of the blocks. DWT is performed on $C_{se\_b}\left(i\right)$ to obtain four matrices for each image block as $C_{se\_b}LL\left(i\right)$, $C_{se\_b}LH\left(i\right)$, $C_{se\_b}HL\left(i\right)$ and $C_{se\_b}HH\left(i\right)$, i=1,⋯,T.(2): DWT is performed again for all matrices $C_{se\_b}LH\left(i\right)$ and $C_{se\_b}HL\left(i\right)$ in (1), respectively. obtaining the matrices $C_{se\_b}LL1\left(i\right)$, $C_{se\_b}LH1\left(i\right)$, $C_{se\_b}HL1\left(i\right)$, $C_{se\_b}HH1\left(i\right)$ and $C_{se\_b}LL2\left(i\right)$, $C_{se\_b}LH2\left(i\right)$, $C_{se\_b}HL2\left(i\right)$, $C_{se\_b}HH2\left(i\right)$, i=1,⋯,T.(3): According to Equations 16 and 17, calculate the energy of each secret image block to generate the average sum of $C_{se\_b}LH1\left(i\right)$, $C_{se\_b}HL1\left(i\right)$, $C_{se\_b}LH2\left(i\right)$ and $C_{se\_b}HL2\left(i\right)$ matrices and denoted as $E_{\mathrm{se}\_b}\left(i\right)$, i=1,⋯,T.
(16)$$\bar{C_{\mathrm{se}\_b}}\left(i\right)=\frac{1}{4}\left(C_{se\_b}LH1\left(i\right)+C_{se\_b}HL1\left(i\right)+C_{se\_b}LH2\left(i\right)+C_{se\_b}HL2\left(i\right)\right),i=1,\cdots ,T,$$
(17)$$E_{\mathrm{se}\_b}\left(i\right)=\sqrt{{\left(\sum \sum \bar{C_{\mathrm{se}\_b}}\left(i\right)\right)}^{2}},i=1,\cdots ,T,$$(4): The four matrices LH1, HL1, LH2 and HL2 are divided into non-overlapping blocks, all blocks are denoted by $D_{M\_b}\left(j\right),j=1,\cdots ,V$, and *V* is the sum of matrix blocks. The matrix block with $j=1,\cdots ,\frac{1}{4}V$ belongs to LH1, $j=\frac{1}{4}V+1,\cdots ,\frac{2}{4}V$ belongs to HL1, $j=\frac{2}{4}V+1,\cdots ,\frac{3}{4}V$ belongs to LH2 and $j=\frac{3}{4}V+1,\cdots ,V$ belongs to HL2.(5): According to Equation (18), calculate the energy of each matrix block $D_{M\_b}\left(j\right),j=1,\cdots ,V$ in (4), denoted by $E_{M\_b}\left(j\right),j=1,\cdots ,V$.
(18)$$E_{M\_b}\left(j\right)=\sqrt{{\left(\sum \sum \bar{D_{M\_b}}\left(j\right)\right)}^{2}},j=1,\cdots ,V,$$(6): After sorting the values in $E_{se\_b}\left(i\right)i=1,\cdots ,T$ and $E_{M\_b}\left(j\right),j=1,\cdots ,V$ in descending order, two sets of image block position indexes $Id_{se\_b}\left(i\right)i=1,\cdots ,T$ and $Id_{M\_b}\left(j\right),j=1,\cdots ,V$ (*V*) can be obtained. The first *T* indexes in $Id_{M\_b}$ are chosen to form a set of mappings with $Id_{se\_b}$, denoted as $EM\{\left(Id_{se\_b}\left(i\right)\right)\to \left(Id_{M\_b}\left(i\right)\right)\},i=1,\cdots ,T$. According to EM and Equation (19), the secret image block $C_{se\_b}$ is embedded into $D_{M\_b}$ of the carrier image.
(19)$$D_{M\_b}\left(Id_{M\_b}\left(i\right)\right)=D_{M\_b}\left(Id_{M\_b}\left(i\right)\right)+a\times C_{se\_b}\left(Id_{se\_b}\left(i\right)\right),i=1,\cdots ,T,$$(7): The modified matrix blocks are merged in $D_{M\_b}$ at $\frac{1}{4}V$ intervals and the merged matrices are $LH1^{\prime }$, $HL1^{\prime }$, $LH2^{\prime }$, $HL2^{\prime }$.

### 4.3. Decryption Algorithm

**Figure 4:**
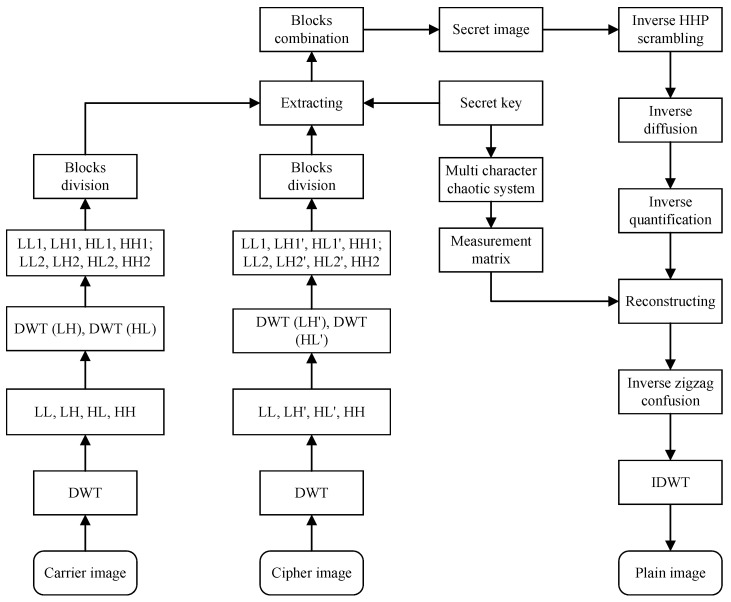
Decryption flow chart of the proposed algorithm.

Decryption algorithm is the inverse operation of encryption algorithm, and consists of two stages. the first stage, the secret image is extracted from visual security cipher image. In the second stage, the decrypted image is reconstructed from the secret image. Before decryption, the sender needs to send the key to the receiver in advance. In addition, the carrier image is selected from a library of images owned by both the receiver and the sender. The complete decryption process is shown in Figure 4.

#### 4.3.1. Extract Secret Images from Visually Secure Cipher Images

**Step 1:** Perform DWT on the cipher image $C_{en}$ to obtain four matrices LL, LH, HL, HH. Then perform DWT transform on the matrices LH and HL, respectively, to obtain LL1, LH1, HL1, HH1 and LL2, LH2, HL2, HH2. The four materices LH1, HL1, LH2 and HL2 are divided into non-overlapping blocks, and the blocks are recorded as $C_{en\_b}\left(j\right),j=1,\cdots ,V$.

**Step 2:** The carrier image is pixel-modified according to Equation (15), and the modified image is noted as ND1. The same transformation steps as in step 1 are applied on ND1 to obtain *V* matrix blocks, recorded as $ND1_{b}\left(j\right),j=1,\cdots V$.

**Step 3:** Acquire the embedding location mapping EM from the key and extract the secret image block according to the following formula.

(20) $$C_{se\_b}\left(ID_{se\_b}\left(i\right)\right)=\frac{C_{en\_b}\left(Id_{M\_b}\left(i\right)\right)-ND1_{b}\left(Id_{M\_b}\left(i\right)\right)}{a},i=1,\cdots ,T,$$

**Step 4:** Compose the secret image blocks into a secret image $C_{se}$ of size M×N.

#### 4.3.2. Recover the Plain Image from the Secret Image

**Step 1:** The secret image $C_{se}$ is extracted by two rounds of HHP as described in Section 3.1, and then the extracted array is inverted by scrambling according to the IdR index to obtain 1×MN one-dimensional array P5.

**Step 2:** Apply the inverse diffusion on P5 to obtain a one-dimensional array P4 of size 1×MN according to the following equation.

(21) P4(i)=P5(i)⊕Y1(i)⊕P5(i−1),i=1,⋯,MN,

Getting P5(0)=floor(AV) from the key, and Y1 is a sequence generated by MCS under key control.

**Step 3:** Reshape P4 into a two-dimensional matrix of size M×N and the P4 matrix is inverse quantized according to the following equation.

(22) $$P3\left(i,j\right)=\frac{P4\left(i,j\right)\times \left(P3_{\max}-P3_{\min}\right)}{255}+P3_{\min},$$

where P3(i,j) and P4(i,j) denote the elements of the respective *i*-th row and *j*-th column, and $P3_{\max}$ and $P3_{\min}$ are obtained from the key.

**Step 4:** The optimization measurement matrix is generated using MCS and SVD as described in Section 4.1.2. Recover the matrix P2 using the OMP algorithm.

**Step 5:** Apply the inverse zigzag obfuscation to the P2 matrix to obtain the sparse coefficient matrix P1. Finally, applying the inverse DWT to P1 results in the plain image *P*. The complete decryption process ends here.

## 5. Simulation Results

In this section, the effectiveness and reliability of the proposed encryption scheme is verified on a laptop computer with a 2.9GHz CPU, 8GB RAM and a Win10 operating system. The relevant parameters involved are as follows: t1=−0.1474, t2=0.1933, t3=0.227, t4=0.4133, sampling distance d=16, threshold TS=30, compression rate CR=0.5, block size 32×32, embedding strength a=0.1 and embedding location mapping EM. The carrier image DWT uses the Haar integer wavelet function, and the compressed sensing reconstruction uses the OMP algorithm.

**Figure 5:**
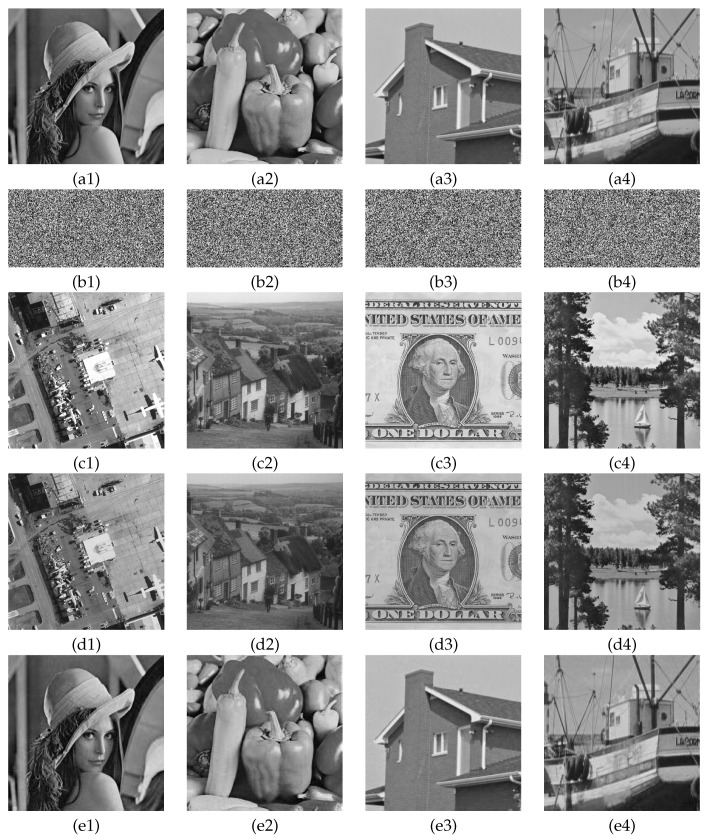
Simulation results of encryption and decryption: (**a1**–**a4**) are plain images Lena, Pepper, House and boat; (**b1**–**b4**) are the corresponding secret images; (**c1**–**c4**) are Carrier images Airfield, Goldhill, Dollar and Boat; (**d1**–**d4**) are the corresponding cipher images; (**e1**–**e4**) are the decrypted images.

The selected test images including size of 256×256 plain images Lena, Pepper, House, Boat, and size of 512×512 carrier images Airfield, Goldhill, Dollar, Sailboat. Figure 5 shows the simulation results of four groups of images. From the first line to the fifth line are plain images, images, carrier images, cipher images and decrypted images. From a visual point of view, the noise-like secret image shown in Figure 5(b1–b4) cannot provide any content information about the plain image, and also compresses the plain image to 1/2 of its original size. Figure 5(d1–d4) generate visually secure cipher images for the secret images embedded into the corresponding carrier images. The cipher image in appearance has the same quality effect as the carrier image shown in Figure 5(c1–c4), which hides the presence of the secret image when transmitted for storage in the Internet and increases the security of the encryption algorithm. Figure 5(e1–e4) are the corresponding decrypted images with better quality. The simulation results show that the proposed algorithm successfully compress, encrypt and embed plain images with excellent encryption and decryption results.

To more visibly assess the imperceptibility and decryption effectiveness, peak signal to noise ratio (PSNR) values and structural similarity (SSIM) values are used to quantitatively analyze image quality [36]. The peak signal-to-noise ratio is the most commonly used method for objectively evaluating image quality. A higher PSNR value indicates a minimum variation between the two images and a better reconstruction accuracy, and the PSNR is calculated as follows.

(23) $$PSNR=10\times \log_{10}\frac{255\times 255}{\sqrt{MSE}},$$

The mean square error MSE calculation is as follows.

(24) $$MSE=\frac{1}{M\times N}\sum_{i=1}^{M}\sum_{j=1}^{N}{\left[X(i,j)-Y(i,j)\right]}^{2},$$

where M×N denotes the image size, X(i,j) and Y(i,j) denote the pixel values at position (i,j) for the plain image and decrypted image, respectively.

Structural similarity is an essential indicator of the similarity of two images. The higher the SSIM value, the more similar of two images will be. The formula of SSIM is shown below.

(25) $$\left\{\begin{array}{c} l\left(X,Y\right)=\frac{2\mu_{X}\mu_{Y}+C_{1}}{\mu_{X}^{2}+\mu_{Y}^{2}+C_{1}}\\ c\left(X,Y\right)=\frac{2\sigma_{XY}+C_{2}}{\sigma_{X}^{2}+\sigma_{Y}^{2}+C_{2}}\\ s\left(X,Y\right)=\frac{\sigma_{XY}+C_{3}}{\sigma_{X}\sigma_{Y}+C_{3}}\\ SSIM(X,Y)=l(X,Y)\times c(X,Y)\times s(X,Y) \end{array}\right.,$$

where $\mu_{X}$ and $\mu_{Y}$ denote the mean values of the plain image *X* and the decrypted image *Y*, respectively, $\sigma_{X}$ and $\sigma_{Y}$ denote the variance of *X* and *Y*, respectively, $\sigma_{X}Y$ denotes the covariance of *X* and *Y*, and $C_{1}$, $C_{2}$, $C_{3}$ are constants.

The PSNR and SSIM results of the cipher and decrypted images in Figure 5 are shown in Table 1. Comparing the data in the first and third columns of Table 1, the PSNR values of the visually significant cipher images are all greater than 44dB and the SSIM values are all greater than 0.97, reflecting the imperceptibility of the proposed algorithm to the embedding of secret images. When comparing the data in the second and fourth columns of Table 1, all the PSNR values of the decrypted images are over 36dB, and the SSIM values of the decrypted images are close to 1, and they are similar to those of plain images. Therefore, the quality of the decrypted images is superior and the proposed algorithm satisfies the requirements of compression and encryption.

## 6. Performance Analyses

### 6.1. Key Space Analysis

The key space refers to the total number of different keys used in the encryption algorithm. The larger the key space, the stronger the ability of the encryption algorithm to resist violent attacks. The violent attack means that the intercepted cryptographic image is cracked with all potential keys in turn until the real correct key is found. Literature [37] points out that the key space required by the encryption algorithm to effectively resist violent attacks is at least $2^{100}$. The key of the encryption scheme proposed in this paper involves (1) mean value AV and standard deviation STD of plain image, (2) External parameters t1, t2, t3 and t4, (3) the chaotic sequence sampling distance *d*, and (4) the secret image blocks embedding location mapping EM. When CR is 0.125, EM has 8 sets of mappings. The higher the CR, the more numbers in EM. The key space of the proposed algorithm in this paper is at least $10^{210}>2^{697}$ when the computer accuracy is $10^{-14}$, and this value is much larger than $2^{100}$. The key space of the proposed encryption algorithm is large enough to effectively resist brute force attacks and has high level of security.

### 6.2. Key Sensitivity Analysis

**Figure 6:**
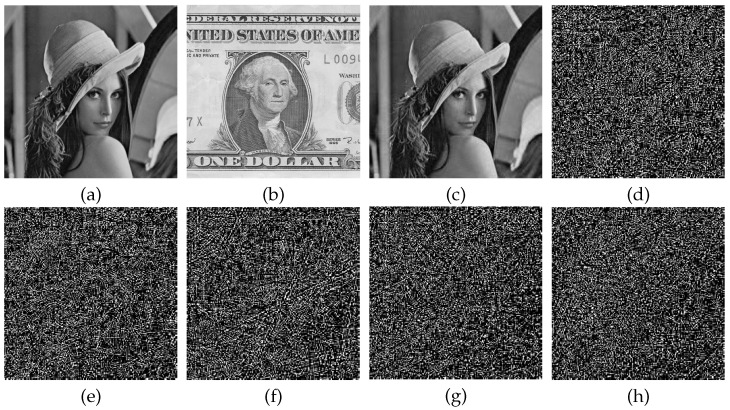
Key sensitivity analysis: (**a**) The plain image Lena; (**b**) The carrier image Dollar; (**c**) Decryption image with the correct key; (**d**) Decryption image of $t1+10^{-14}$; (**e**) Decryption image of $t2+10^{-14}$; (**f**) Decryption image of $t3+10^{-14}$; (**g**) Decryption image of $t4+10^{-14}$; (**h**) Decryption image of d+1.

Secure cryptosystems require a high sensitivity to the key during encryption decryption, where a micro change in the key results in a dramatic change in the decrypted image. In this section, size of 256×256 Lena is selected as the plain image and size of 512×512 Dollar as the carrier image. The sensitivity in the decryption process is verified by changing the key t1, t2, t3 and t4 by $10^{-14}$ magnitude and changing the sampling distance *d* by 1 step. For the current key modification, the other key parameters are constant. Figure 6 shows the results of the key sensitivity test for the decryption process. As can be seen in Figure 6, once the decryption key changed, the recovered decrypted image without any valid information is completely different from the image decrypted with correct key. Hence, the decryption process of the algorithm is highly sensitive to the key.

Pixel change rate (NPCR) is introduced to measure the ratio of different grey values at the same position in the image [38]. NPCR of decryption images with different keys changed in Figure 6d–h and decryption images with correct keys in Figure 6c are shown in Table 2. Over 99% of the pixels in the decrypted image are altered when the key is changed extremely slightly during the decryption process. An indication that the algorithm is highly sensitive to the key during the decryption process.

### 6.3. Correlation Analysis

**Figure 7:**
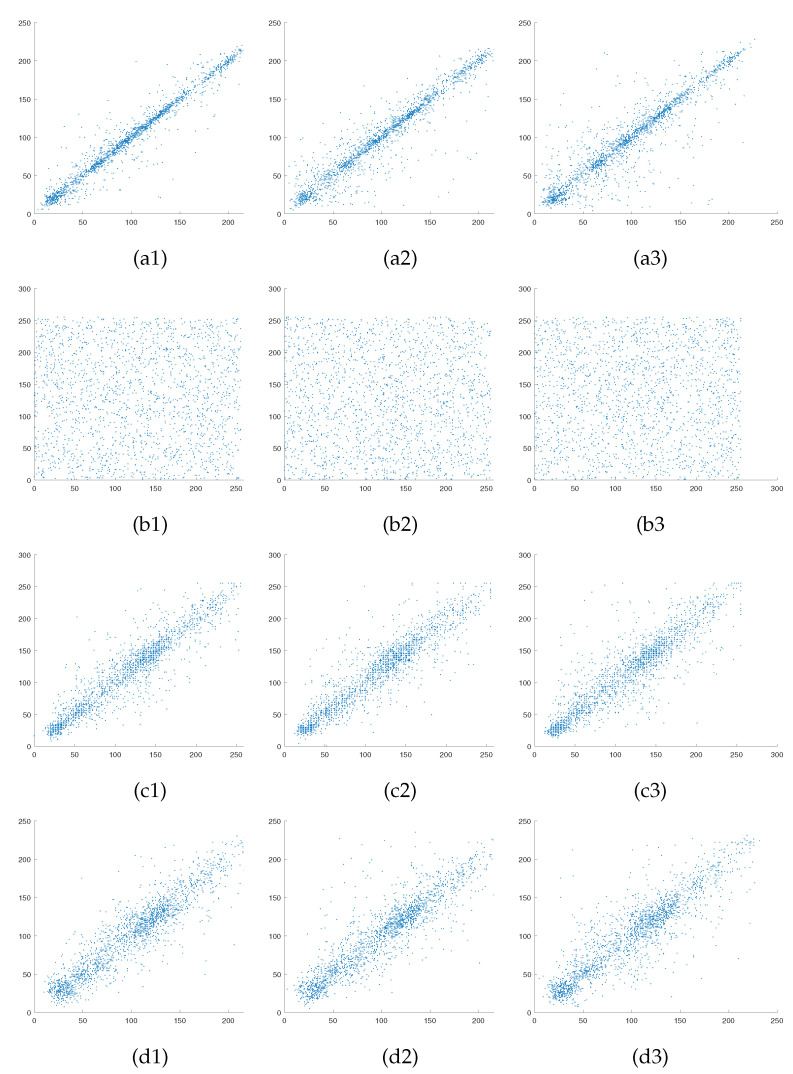
Pixel correlation analysis: (**a1**–**a3**) are correlation of the plain image Lena in horizontal, vertical and diagonal directions; (**b1**–**b3**) are correlation of the secret image in horizontal, vertical and diagonal directions; (**c1**–**c3**) are correlation of the carrier image in horizontal, vertical and diagonal directions; (**d1**–**d3**) are correlation of cipher in horizontal, vertical and diagonal directions.

Adjacent pixel correlation reflects the correlation degree of pixel values at adjacent locations in an image. A successful image encryption algorithm should have the ability to reduce the correlation of adjacent pixels. Pixel correlation analysis is generally performed in three directions: horizontal, vertical and diagonal from the image. Our experiments are conducted with size of 256×256 Lena as the normal image and size of 512×512 Airfield as the carrier image, and 2000 pairs of adjacent pixels are randomly selected for correlation analysis. The pixel correlation in the three directions is shown in Figure 7. Besides, the correlation coefficient is used to measure the degree of correlation between image pixels [39]. The correlation coefficient is calculated as shown following, and the specific correlation coefficient values are shown in Table 3.

(26) $$\rho_{xy}=\frac{E\left\{\left[x-E(x)][y-E(y)\right]\right\}}{\sqrt{D(x)}\sqrt{D(y)}},$$

where *x* and *y* denote adjacent pixels, and E(x) and E(y) denote the expectation and variance, respectively. For images, the correlation coefficient takes values in the range [0,1]. The value of 0 indicates no correlation, and value of 1 indicates full correlation. From Figure 7(a1–a3) and the first row of Table 3, a strong correlation can be observed in the linear arrangement of adjacent pixel distributions in all three directions of the plain image, with correlation coefficients greater than 0.9 close to 1. From Figure 7(b1–b3) and the second row of Table 3, the adjacent pixel distributions of the secret images are scattered and the correlation coefficients are all less than 0.01 close to 0 and not correlated. Furthermore, comparing Figure 7(c1–c3) and (d1–d3) and the third four rows of Table 3, the pixel distribution trends in all directions for the encrypted and carrier images are approximately the same, with very close correlation coefficients. Experiment results show that the proposed encryption algorithm with good security in this paper greatly reduces the correlation between the secret image pixels and guarantee the cipher image pixel correlation and carrier image is highly similar.

**Figure 8:**
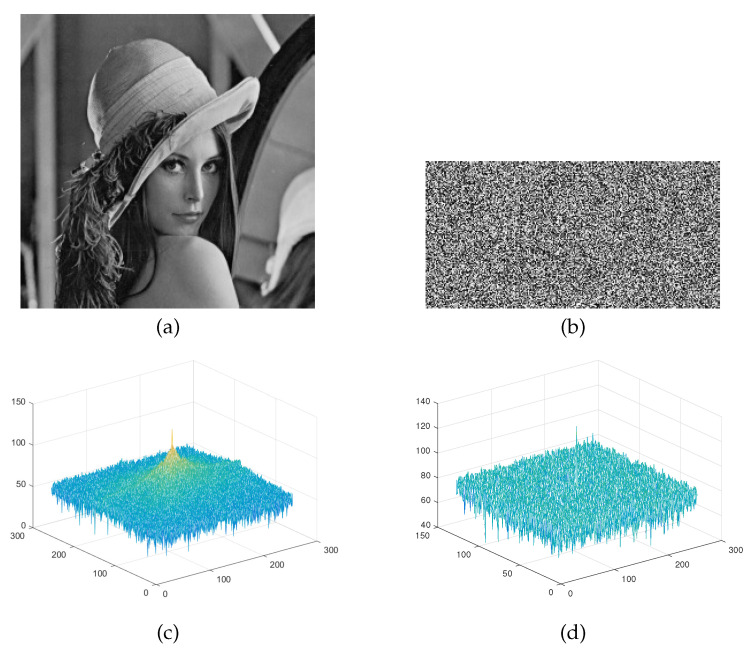
Power spectral density: (**a**) The plain image Lena; (**b**) Lena’s secret image; (**c**) Plain image power spectrum (**d**) Secret image power spectrum.

The power spectrum density curve of an image characterizes the distribution of image power in the frequency domain. The power spectrum of an image is defined by the following equation.

(27) $$P\left(s\right)={\left|F\left[s(L,H)\right]\right|}^{2},$$

where P(s) denotes the power spectrum of image *s*, *F* is the Fourier transform, and *L* and *H* are the height and width of the image, respectively. Figure 8 shows the power spectral density maps of the 256×256 Lena plain image and its secret image. It can be seen that the power spectral density map of the plain image has a wavy conical shape, while the power spectral density map of the secret image is almost flat and uniform. Therefore, the secret image generated by the proposed encryption algorithm is almost uncorrelated with the plain image.

### 6.4. Histogram Analysis

**Figure 9:**
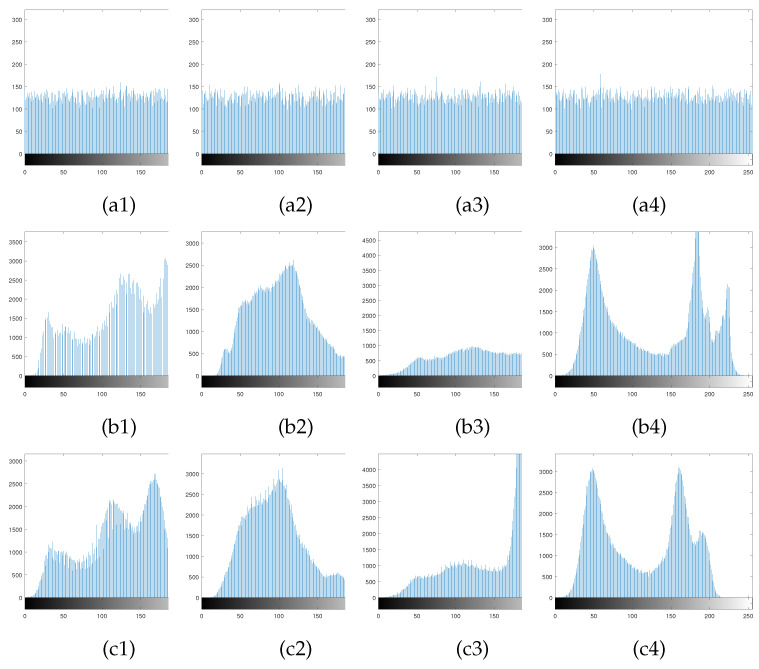
Histogram analysis: (**a1**–**a4**) are the corresponding secret image histograms of the plain images Lena, Pepper, House and Boat; (**b1**–**b4**) are histogram of the Carrier images Airfield, Goldhill, Dollar and Boat, respectively; (**c1**–**c4**) are histogram of the corresponding cipher images.

A histogram is a graphical representation of the intensity distribution of pixels in an image. It counts the number of pixels for each intensity value, but does not contain information regarding the position of these pixels in the image [40]. The test image set is the same as in Section 5, applying encryption and decryption to the four plain images, and Figure 9 shows the histograms of the secret image, carrier image and cipher image. In Figure 9, it can be seen the histogram of the secret image is flat and more evenly distributed, which indicates that it is less susceptible to attack. Histograms of the cipher images have approximately the same distribution as the histograms of the carrier images, indicating that the embedding of the carrier images hardly corrupts the information in the carrier images.

The variance of a histogram measures the range of fluctuations in the frequency of each pixel value, and the standard deviation of a histogram reflects the degree of dispersion in the frequency of each pixel value. The variance of a histogram is calculated by the following formula [41].

(28) $$D=\frac{1}{256}\sum_{i=0}^{255}[x_{i}-{\left(\frac{L\times H}{256}\right)}^{2},$$

where *D* is the variance of the histogram, *x* is the frequency of each pixel value in the histogram, *L* is the height of the image and *H* is the width of the image. The larger the histogram variance, the more pronounced the histogram fluctuations, and the smaller the variance, the flatter the fluctuations.

The standard deviation of the histogram is calculated as shown in the following.

(29) $$\sigma =\sqrt{D},$$

where σ is the standard deviation of the histogram. Lower standard deviation indicates a more uniform frequency distribution of each pixel value. Table 4 shows the variance and standard deviation of the four plain images and their secret images. The variance and standard deviation of the plain images are much higher than those of the secret images, indicating that the secret image histograms have excellent uniformity. In addition, using a size of 256×256 Lena as the test image, we compared the variance and standard deviation of the histogram with the Ref. [41]. As can be seen from Table 5, the variance and standard deviation of the histogram in this paper are lower than that of the offering, indicating the excellent performance of the proposed algorithm in encryption.

### 6.5. Information Entropy

The entropy in information theory represents the average amount of information output by the source, and the magnitude of the entropy is a measure of the average uncertainty and complexity of the signal [42]. The entropy H(s) of an information source *s* is calculated as shown in the following formula.

(30) $$H\left(s\right)=-\sum_{i=0}^{N}p\left(s_{i}\right)\log_{2}p\left(s_{i}\right),$$

where $p(s_{i})$ denotes the probability of occurrence of $s_{i}$ and *N* denotes the total number of $s_{i}$. In the ideal case for a grayscale image, where all grayscale values in the image occur with equal probability, the information entropy of the image is equal to 8 [43]. Selecting the same test images as in Section 5 for encryption and decryption, information entropy analysis is performed on the corresponding resulting secret image and cipher images. Table 6 lists the information entropy results corresponding to the four sets of test images, which shows the information entropy of the secret image to be close to 8, demonstrating that all pixel values of the secret image occur with equal probability. The entropy values of the carrier image and the cipher image are close to each other, which indicates that the embedded secret image is undetectable. The decrypted image and the plain image have similar entropy values, indicating that the image reconstruction is better. In summary, the encryption scheme is secure against entropy attacks.

### 6.6. Known Plaintext Attack and Chosen Plaintext Attack

Currently, several preexisting encryption algorithms have been broken by known plaintext attack (KPA) and chosen plaintext attack (CPA). The encryption algorithm in this paper is divided into two stages. In the phase of generating noise-like secret images, the mean AV and standard deviation STD of the plain images are calculated to determine the initial values of the chaotic system, and the chaotic sequence generated by the chaotic system is used to design the measurement matrix for compression perception. It means that the measurement matrix varies with the plain image, ensuring the security of the encryption algorithm. Besides this, the random sequence generated by the chaotic system also serves for scrambling and diffusion operations, and the resulting secret image is related to the plain image. When different images are encrypted, the corresponding keys change. The attacker cannot obtain useful information by encrypting certain special images. In the phase of generating visually secure cipher images, the embedding position of the secret image block is related to the secret image and the carrier image, and the secret image relates to the normal image. Therefore, in this paper, the embedding method improves the randomness of the embedding result and provides more security to the encryption algorithm. In summary, both phases of the encryption algorithm proposed in this paper are relevant to plain images and can better resist KPA and CPA.

### 6.7. Robustness

During transport over network channels, cipher images may be polluted by noise or clipping under natural or artificial circumstances, which can affect the quality of image reconstruction. To test the impact of these interferences on the quality of the decrypted images, size of 256×256 Lena is selected as the normal image and size of 512×512 Airfield as the carrier image to analyze the robustness of the encryption algorithm.

#### 6.7.1. Noise Attack

**Figure 10:**
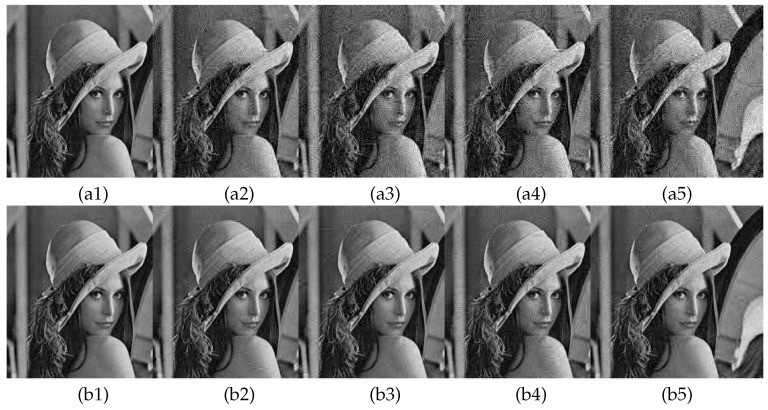
Noise attack analysis. (**a1**–**a5**) are decrypted images with GN intensities of 0.000001%, 0.000003%, 0.000005%, 0.000007%, and 0.000009%, respectively; (**b1**–**b5**) are decrypted images with SPN intensities of 0.001%, 0.003%, 0.005%, 0.007%, and 0.009%, respectively.

Gaussian noise (GN) and salt and pepper noise (SPN) are added separately to the cipher image. Figure 10(a1–a5) show the corresponding decoded images when Gaussian noise strengths of 0.000001%, 0.000003%, 0.000005%, 0.000007% and 0.000009% are added. Table 7 shows the decrypted image PSNR values in Figure 10. As can be seen in Figure 10(a1–a5) and Table 7, the decryption quality decreases as the Gaussian noise intensity increases, but its PSNR value is still above 29dB. Figure 10(b1–b5) show the corresponding decrypted images when adding pretzel noise intensities of 0.001%, 0.003%, 0.005%, 0.007% and 0.009%. Table 8 shows the PSNR values of the decrypted images in Figure 10(b1–b5). From Figure 10(b1–b5) and Table 8, the decrypted image quality is inversely related to the addition of pretzel noise density, but the PSNR value of the decrypted image is still calculated to be above 33dB. Comprehensive above shows that the encryption algorithm in this paper has certain robustness to noise.

#### 6.7.2. Data Cropping Attacks Analysis

**Figure 11:**
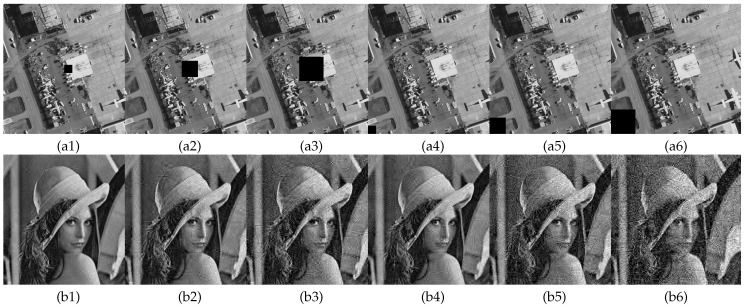
Data cropping attack analysis: (**a1**–**a3**) are the cipher images center cropped to 32×32, 64×64, 96×96; (**a4**–**a6**) are the cipher image edge cropped to 32×32, 64×64, 96×96; (**b1**–**b6**) are the decryption images corresponding to (a1–a6).

Perform central data cropping and edge data cropping attacks on cipher image with crop sizes of 32×32, 64×64, 96×96. Figure 11(a1–a3) shows the cipher image central data loss and Figure 11(a4–a6) shows the cipher image edge data loss. Figure 11(b1–b6) show the corresponding decoded images for Figure 11(a1–a6). As can be seen in Figure 11, as the range of data loss becomes larger, the corresponding quality of the decrypted images decreases significantly in visual effect. Moreover, different locations of data loss correspond to different quality of the decrypted image, but all of them can observe plain image information from the decrypted image. Thus, the encryption algorithm in this paper has better resistance to data cropping.

### 6.8. Compression Capability Analysis

**Figure 12:**
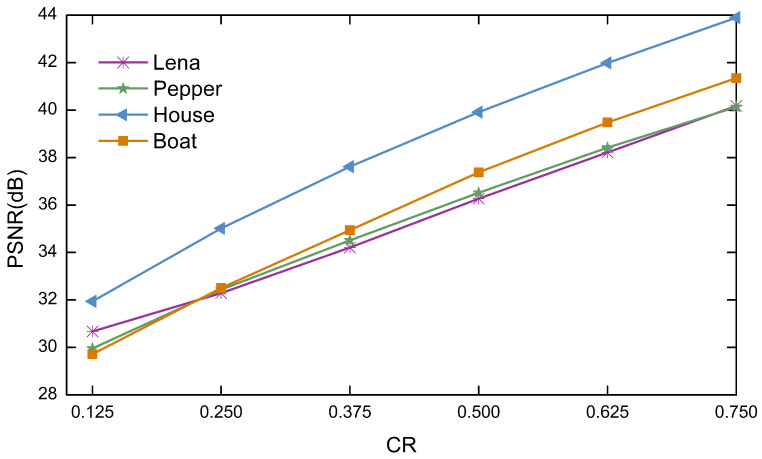
Relationship between different compression ratio and reconstruction quality.

In this section, to analyze the effect of different compression ratios on the quality of the image reconstruction. The plain images are selected as Lena, Pepper, House, Boat of size 256×256, and the carrier image is selected as Airfield of size 512×512. The compression ratios of the plain images are set to 0.125, 0.25, 0.375, 0.5, 0.625 and 0.75 in that order. Figure 12 shows the relationship between different compression ratios and the PSNR of the reconstructed images. Observation shows that the PSNR increases with increasing CR, that is, the higher the compression rate, the better the reconstruction effect. When CR=0.125, the PSNR values for Lena and House are 30.6735dB and 31.943dB, illustrating that the algorithm in this paper can recover superior image quality even at a very small compression rate. When CR≥0.25, the reconstruction quality of the tested images is all greater than 32dB. Table 9 shows the reconstructed PSNR compared to other algorithms at different compression ratios, where the size of the normal image is 256×256. When CR=0.25, the PSNR values of the proposed algorithm are significantly better than that in Refs. [44,45,46]. When CR=0.5, the PSNR value of the proposed algorithm is at least 5.3426dB higher than that of the algorithms in Refs. [44,45,46,47,48]. Consequently, the algorithm proposed in this paper has excellent compression performance and can be used for secure communication to satisfy different needs.

### 6.9. Complexity

The time complexity of the algorithm qualitatively describes the operational efficiency of the algorithm. Assume that the size of the plain image is N×N, the compression rate is CR, and the sampling interval of the chaotic system is *d*. The time complexity of generating the four chaotic sequences is O(1000+CR×N×N×d), the time complexity of constructing the measurement matrix is O(CR×N×N), the time complexity of the clockwise print placement is O(1/4×CR×N×N), and the time complexity of the diffusion is O(CR×N×N), so the total time complexity of the compression and encryption phase is O(CR×N×N). The time complexity of the embedding stage mainly depends mainly on the total number of blocks of the secret image, and the other parts are performed linearly. Assuming that the total number of blocks of the secret image is *l*, the time complexity of the embedding stage is O(l).

### 6.10. Time Efficiency Analysis

In the actual communication, apart from security, encryption algorithms are needed for faster encryption and decryption of plain images. The processing speed can be described qualitatively in terms of the running time of the algorithm. The encryption and decryption process of the proposed algorithm includes, encryption of the plain image and embedding of the secret image, extraction of the secret image and reconstruction of the plain image. The size of 256×256 Lena is selected as the plain image and size of 512×512 Airfield as the carrier image for testing. Table 10 shows the encryption and decryption times for the different images at different stages, it is evident from this that the encryption process takes 9.31 % of the total time and the decryption process takes 90.69 % of the total time. The plain image encryption phase accounted for 81.78% of the total encryption process time, the secret image embedding carrier image phase accounted for 18.22% of the total encryption process time, the secret image extraction phase occupied 1.54% of the decryption process, and the plain image reconstruction occupied 98.46% of the decryption process. The time consumption of the proposed encryption algorithm is focused on applying chaotic sequence to construct the measurement matrix, permutation, diffusion and reconstruction steps.

## 7. Conclusions

This paper proposes a visually secure image encryption algorithm based on compressed perception and region energy. It can achieve both image data security and appearance security. At the core of the encryption algorithm is to propose a method of HHP scrambling for increasing the randomness between matrix elements. Additionally, a region energy matching embedding is utilized for the encryption process, which vastly reduces the damage to the visual quality of the carrier image. Simulations show that the key space of the proposed algorithm is at least $2^{697}$ and has high key sensitivity, effectively resisting violent attacks. Adjacent pixel correlation and histogram analysis reflect that the algorithm has excellent security and the entropy of the secret image information is close to 8, proving that it is secure against entropy attacks. In addition, the algorithm is also proved to be effective withstand chosen-plaintext attack and known-plaintext attack, noise attacks and data cropping attacks with excellent robustness. Next, we will investigate secure encryption systems with massive embedding and high robustness to improve the security of color image transmission.

## Figures and Tables

**Table 1:** PSNR and SSIM values of cipher images and decryption images.

Plain Image	Carrier Images	PSNR (dB)		SSIM	
		Cipher Image	Decrypted Image	Cipher Image	Decrypted Image
Lena	Airfield	45.9768	36.2680	0.9756	0.9938
Pepper	Goldhill	46.8526	36.5198	0.9748	0.9945
House	Dollar	51.9394	39.9148	0.9740	0.9969
Boat	Sailboat	44.6585	37.3770	0.9768	0.9951

**Table 2:** Decryption image NPCR with different key.

Item	Keys				
	$t1+10^{-14}$	$t2+10^{-14}$	$t3+10^{-14}$	$t4+10^{-14}$	d+1
NPCR	99.6872%	99.7116%	99.7101%	99.6918%	99.7131%

**Table 3:** Correlation coefficients.

Image	Horizontal	Vertical	Diagonal
plain image (Lena)	0.9736	0.9417	0.9178
Secret image	0.0229	−0.0039	−0.0106
Carrier image (Airfield)	0.9442	0.9176	0.8943
Cipher image	0.9356	0.9060	0.8762

**Table 4:** The variance and standard deviation of the histograms of ordinary and secret images.

Item	Plain Image				Secret Image			
	Lena	Pepper	House	Boat	Lena	Pepper	House	Boat
*D*	30,666	36,653	299,789	102,311	127	136	128	128
σ	175	191	548	320	11	12	11	11

**Table 5:** Comparison of standard deviation and variance of histogram.

Item	Plain Image Lena		Lena’s Secret Image	
	Ref. [41]	Ours	Ref. [41]	Ours
*D*	38,451	30,666	414	127
σ	196	175	20	11

**Table 6:** Information entropy.

Plain Image	Carrier Images	Entropy				
		Plain Image	Secret Image	Carrier Image	Cipher Image	Decrypted Image
Lena	Airfield	7.5683	7.9873	7.1206	7.5631	7.5934
Pepper	Goldhill	7.5352	7.9863	7.4778	7.3116	7.5748
House	Dollar	6.4971	7.9860	6.9785	6.9621	6.5918
Boat	Sailboat	7.1456	7.9871	7.4758	7.3538	7.1870

**Table 7:** PSNR values of GN with different intensity.

GN	Noise Intensity				
	0.000001%	0.000003%	0.000005%	0.000007%	0.000009%
PSNR (dB)	34.3698	30.5790	29.9235	29.5931	29.4689

**Table 8:** PSNR values of SPN with different intensity.

SPN	Noise Intensity				
	0.001%	0.003%	0.005%	0.007%	0.009%
PSNR (dB)	35.7661	35.0246	34.9296	34.5806	33.5185

**Table 9:** PSNR comparison.

CR	Plain Image	PSNR (dB)					
		Ref. [48]	Ref. [44]	Ref. [47]	Ref. [45]	Ref. [46]	Ours
0.25	Lena	-	<29	-	24.39	<23.3846	32.290
	House		<30		-	-	35.0177
0.5	Lena	30.0233	<31	23.3608	30.71	27.7247	36.268
	House	34.5722	<34	-	-	-	39.9148
	Pepper	29.8787	-	27.3366			36.5198

**Table 10:** Time consumed for encryption and decryption.

Item	Time (s)
Compressing	0.266066
Embedding	0.059261
Total Encryption	0.325327
Extracting	0.048770
Reconstruction	3.119658
Total Decryption	3.168428
Total	3.493755

## Data Availability

Not applicable.
